# The Genetics of Human Schistosomiasis Infection Intensity and Liver Disease: A Review

**DOI:** 10.3389/fimmu.2021.613468

**Published:** 2021-02-15

**Authors:** Estelle M. Mewamba, Oscar A. Nyangiri, Harry A. Noyes, Moses Egesa, Enock Matovu, Gustave Simo

**Affiliations:** ^1^Molecular Parasitology and Entomology Unit, Faculty of Science, University of Dschang, Dschang, Cameroon; ^2^College of Veterinary Medicine Animal Resources and Biosecurity, Makerere University, Kampala, Uganda; ^3^Centre for Genomic Research, School of Biological Sciences, University of Liverpool, Liverpool, United Kingdom; ^4^Medical Research Council/Uganda Virus Research Institute and London School of Hygiene & Tropical Medicine Uganda Research Unit, Entebbe, Uganda; ^5^London School of Hygiene and Tropical Medicine, London, United Kingdom

**Keywords:** schistosomiasis, fibrosis, Th17, intensity of infection, QTL, linkage

## Abstract

Schistosomiasis remains the fourth most prevalent parasitic disease affecting over 200 million people worldwide. Control efforts have focussed on the disruption of the life cycle targeting the parasite, vector and human host. Parasite burdens are highly skewed, and the majority of eggs are shed into the environment by a minority of the infected population. Most morbidity results from hepatic fibrosis leading to portal hypertension and is not well-correlated with worm burden. Genetics as well as environmental factors may play a role in these skewed distributions and understanding the genetic risk factors for intensity of infection and morbidity may help improve control measures. In this review, we focus on how genetic factors may influence parasite load, hepatic fibrosis and portal hypertension. We found 28 studies on the genetics of human infection and 20 studies on the genetics of pathology in humans. *S. mansoni* and *S. haematobium* infection intensity have been showed to be controlled by a major quantitative trait locus *SM1*, on chromosome 5q31-q33 containing several genes involved in the T_h_2 immune response, and three other loci of smaller effect on chromosomes 1, 6, and 7. The most common pathology associated with schistosomiasis is hepatic and portal vein fibroses and the *SM2* quantitative trait locus on chromosome six has been linked to intensity of fibrosis. Although there has been an emphasis on T_h_2 cytokines in candidate gene studies, we found that four of the five QTL regions contain T_h_17 pathway genes that have been included in schistosomiasis studies: *IL17B* and *IL12B* in *SM1, IL17A* and *IL17F* in 6p21-q2, *IL6R* in 1p21-q23 and *IL22RA2* in *SM2*. The T_h_17 pathway is known to be involved in response to schistosome infection and hepatic fibrosis but variants in this pathway have not been tested for any effect on the regulation of these phenotypes. These should be priorities for future studies.

## Introduction

Schistosomiasis is caused by digenic trematodes of the genus *Schistosoma* with *Schistosoma mansoni* and *Schistosoma haematobium* causing the majority of human infections. Adult parasites live in the veins around the gut and bladder and eggs are excreted in feces or urine and infect snails in fresh water. Parasite numbers are amplified in the snail intermediate host and human infective stages then emerge that can penetrate human skin when people enter the water. Schistosomiasis induces acute, severe, and chronic morbidity among those who are infected and can cause liver and bladder fibrosis and eventually bladder or colorectal cancer (1). Although exposure to water infested with the infective schistosome cercariae is the main risk factor for schistosomiasis there is considerable variation in infection intensity between people with similar exposures and schistosomiasis cases aggregate in families, some of this variation has been attributed to the genetics of the human immune response (2–4).

A review of the genetics of human susceptibility to schistosomiasis related fibrosis has been published recently (5), but there has been no review of the genetics of schistosome infection since 2008 (2).

A fundamental understanding of the genetics of schistosomiasis susceptibility and high worm load may contribute to rational design of interventions, including vaccines (6). For example, it has recently been shown that a set of 32 SNPs in 10 genes can predict susceptibility to severe hepatic disease among Brazilians with 63% sensitivity and 90% specificity (5). In the present review of the genetics of human susceptibility to schistosomiasis we focus on loci associated with egg/worm burden and hepatic fibrosis.

We therefore present an updated review of the genes and variants that have been found associated with schistosomiasis infection intensity and liver disease, together with a review of genes within QTL that could be prioritized for future analyses. We have excluded the HLA region since we have only identified one study of genes in this region since they were was last reviewed (7, 8).

### Epidemiology and Treatment

The disease affects almost 240 million people, and 700 million are at risk of infection in 74 countries, the majority being in Africa, Asia and South America (9, 10). Between 3 and 56 million disability-adjusted life years (DALYs) are lost per annum and 280,000 deaths per annum have been attributed to effects of schistosomiasis (11–13). Approximately 85% of infections occur in sub-Saharan Africa and at least 90% of people requiring treatment for schistosomiasis live in Africa (14). Schistosomiasis can also be associated with chronic anemia, childhood growth stunting, protein calorie malnutrition, cognitive disability, and poor school performance (15–20).

Control of schistosomiasis has continued to rely mainly on mass drug administration (MDA) of school-aged children using the anti-schistosomal drug praziquantel (PZQ) (21, 22). Although this strategy has reduced morbidity, the impact on prevalence has been more limited, because praziquantel does not kill immature schistosomes (23, 24), coverage remains restricted and only school age children are routinely treated. Vaccines are in development but phase three trials have not been successful (25).

### Immune Responses During Schistosome Infections

Immune responses to penetrating and migrating *Schistosoma* larvae (schistosomula) and maturing adults are predominantly T_h_1 (26). Excretory/secretory *Schistosoma* antigens damage host barrier cells which release alarmins, activate innate cells and induce proinflammatory cytokines (IL1B, IL6, IL17, TNF, and IFNG) (27). About 6 weeks after infection, *Schistosoma* eggs are deposited in tissues (the liver and the intestine or the bladder) and trigger an expansion of T_h_2 cells (28). *Schistosoma* egg antigens also directly bind receptors on antigen presenting cells inhibiting IL12 production and consequently T_h_1 responses (29). T_h_2 responses can also be induced independently of egg deposition as infection with single sex schistosomes induce pre-patent IL4 production by CD4 T cells (30). Schistosome specific T_h_2 responses are downmodulated in long-standing infections (31) and this is associated with a development of regulatory cells producing IL10 and transforming growth factor beta (TGFB). This not only allows the parasite to survive in the host and minimize host tissue damage but also modulates host immune responses to unrelated antigens including allergens, self-antigens, and vaccines.

Schistosome egg secretions are highly antigenic (31) and typically induce polarized granulomatous T_h_2 responses (32). Granulomas form around eggs lodged in tissues to protect tissue cells (33) but persistent host CD4 T_h_2 cell mediated responses to parasite eggs cause fibrosis (34). The pro-fibrotic T_h_2 cytokine IL13 is associated with periportal fibrosis in humans (35). Beyond T_h_2 cytokine responses, intensified hepatic granulomatous inflammation in *S. mansoni* infected mice is associated with high levels of IL17 and controlled by IFNG (36). In human schistosomiasis, IL17 producing CD4 T helper cells are associated with ultrasound textural abnormalities while T regulatory cells are associated with reductions in this pathology (37).

### Phenotypes

#### Schistosomal Fecal Egg Count and Worm Burden

Studies of the human genetics of susceptibility to schistosomiasis have focussed on two classes of phenotype; infection associated phenotypes and pathology related phenotypes. Infection associated phenotypes are usually egg counts or worm burden estimates but sometimes total IgE as a marker of the immune response (38). Eggs counts are obtained by the Kato Katz (KK) method for *S. mansoni* and by urine filtration for *S. haematobium* and worm burdens are estimated by measuring the circulating cathodic antigen (CCA) in urine or circulating anodic antigen (CAA) in plasma (39) that are produced by adult worms.

Approximately 80% of the environmental egg burden from helminths including schistosomes, derives from ~20% of the cases (40). For example 22 out of 119 Kenyan school children had developed high *S. mansoni* egg burden (>100 eggs per gram of feces) 12 months after treatment, whilst 70 children still had low (<30 epg) egg burdens (41) and this effect was not correlated with the amount of water contact. This tendency for some people to develop high infections even after treatment has been attributed to variation in genetic risk (42).

Schistosome egg burdens are also highly skewed by age with egg burdens increasing up to the age of puberty and declining thereafter (43–46). A study in Brazil found that children under 19 had egg burdens that were over ten times higher than older adults (42). The higher intensity and frequency of infections in children may be due to the slow development of immunity to schistosomes. Possibly, the antigens that are exposed by dying worms cross react with larval antigens and stimulate a protective anti-larval IgE response. The long life span of adult worms (5–15 years) means that it takes many years for children to be exposed to sufficient dying worms to develop an IgE response to the larvae (47). High anti-parasite IgE levels have been associated with resistance and high specific IgG4 has been associated with susceptibility and it has been proposed that the ratio of these two immunoglobulins controls resistance to schistosomiasis (48–51).

#### Schistosomiasis Associated Hepatic and Periportal Fibrosis and Portal Vein Hypertension

Although schistosomes cause a wide range of symptoms and fibrotic lesions can form around egg granulomas in many tissues, the main indicator of *S. mansoni* and *S. japonicum* pathology is hepatic fibrosis (HF) and periportal fibrosis (PPF) (52). WHO guidelines provide scoring scales for HF and PPF (53) and both scales are used as phenotypes in genetic research (54). HF and PPF is caused by extracellular matrix forming around schistosome eggs. In the hepatic portal vein this can lead to portal hypertension (PH) (55, 56), ultimately, some patients with PH die of internal bleeding, superinfections, or heart or kidney failure. *S. haematobium* is associated with bladder cancer and *S. mansoni* may be associated with hepatocellular carcinoma (57) but genetic studies of the pathology of schistosomiasis have focussed almost exclusively on hepatic and periportal fibrosis and in this review all references to pathology are to these closely related conditions unless otherwise indicated. Fibrosis can be measured by ultra-sound scan, although there are concerns about the accuracy and reproducibility of ultrasound (58); additional markers and protocols for grading pathology are being developed but are not well-validated (59–61).

## Genome Wide Linkage Studies Discover Schistosomiasis Susceptibility Loci

The reviews of linkage and candidate gene studies of schistosomiasis are broken down into two sections by phenotype: (1) studies of infection status, which is usually determined by egg count in urine or feces and (2) studies of pathology which is mainly periportal fibrosis determined by ultra-sound. Relevant publications were identified by searching PubMed using the terms in Supplementary Table 1.

### Heritability of Schistosomiasis Infection Risk

Heritability, the proportion of risk attributable to genetic variation, must be substantial to be detectable. A summary of heritability estimates for schistosomiasis are shown in Table 1. Studies in Brazil (62–64); Kenya (46); and China (65, 66) have each estimated similar proportions of the variance of *S. mansoni* egg count that are attributable to genetic variation, with additive heritability (*h2*) estimates of 23–31%. However, there were striking differences in the two estimates for heritability of infection with *S. japonicum* in China using variance components (VC) (0 & 58%) (Table 1), which have been discussed by others (2).

**Table 1:** Heritability estimates for genetic component of risk of schistosomiasis in different populations.

**Parasite**	**Phenotype**	**Country and district**	**Heritability estimate and method eg *h2***	**References**
*S. mansoni*	Egg count	Brazil, Minas Gerais	23% (*h2*)	(62)
*S. mansoni*	Egg count	Brazil, Minas Gerais	27% (VC)	(63)
*S. mansoni*	Egg count	Brazil, Bahia	31% (VC)	(64)
*S. mansoni*	IgE	Brazil, Bahia	59% (VC)	(64)
*S. haematobium*	Egg count	Kenya, Coast	9% (*h2*)	(46)
*S. haematobium*	Bladder morbidity	Kenya, Coast	14% (*h2*)	(46)
*S. japonicum*	Infection	China, Jiangxi	58% (VC)	(65)
*S. japonicum*	Infection	China Sichuan	0% (VC)	(66)

*h2 additive heritability, VC variance components*.

#### Linkage Studies for *Schistosoma* Egg Count

The initial study of the genetics of human schistosomiasis used segregation analysis, which determines whether the distribution of the disease on family pedigrees is consistent with the presence of a major gene (3). This study demonstrated the presence of a major gene which was subsequently named *SM1* and located on chromosome five by parametric linkage analysis (4). A major gene has alleles that cause a difference in phenotype between family members that is large enough to be able to categorize individuals as carriers or non-carriers on the basis of phenotype alone (67) and parametric linkage analysis requires estimates of the disease allele frequency and penetrance of the phenotype for the three possible genotypes.

**Figure 1:**
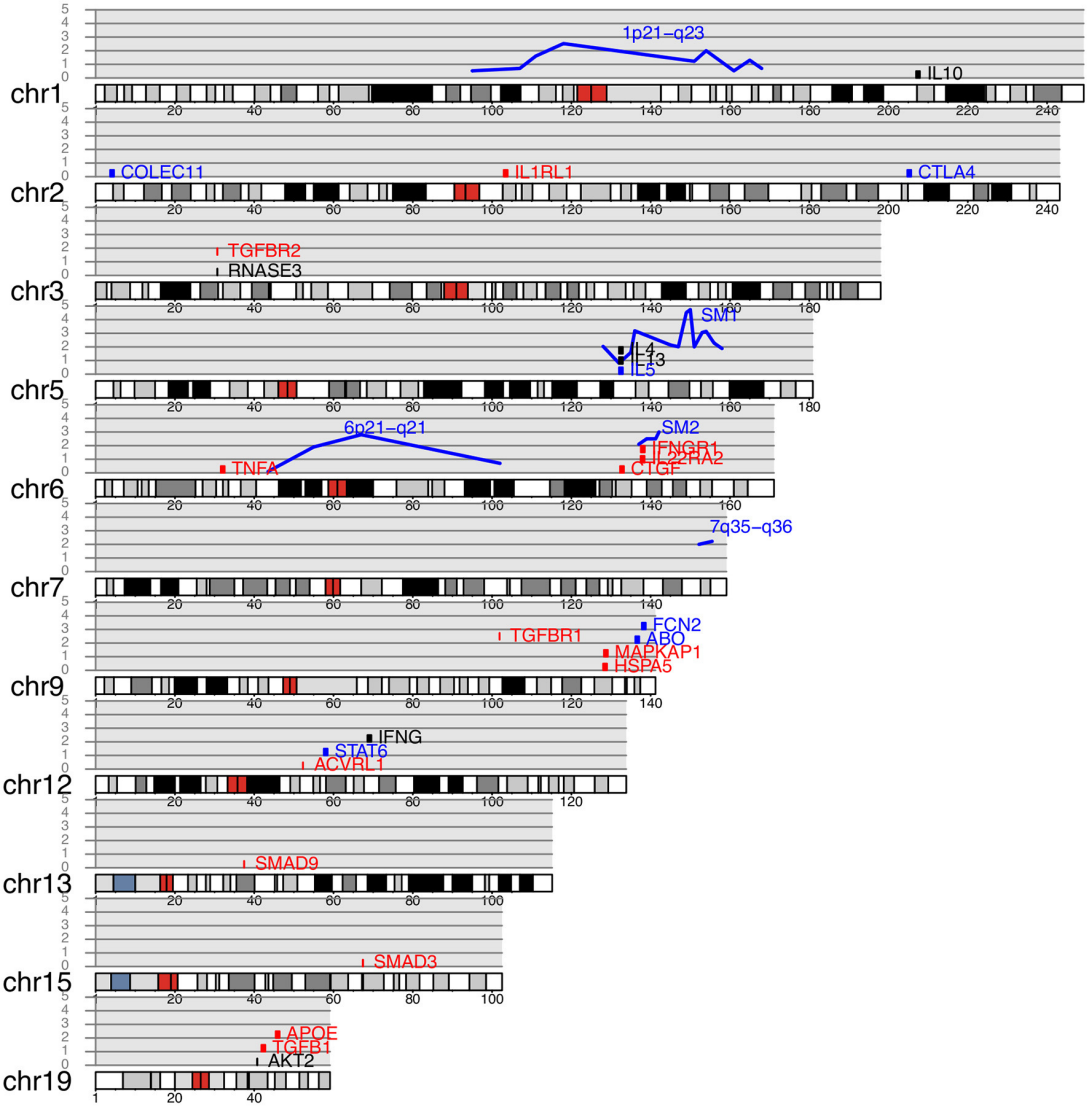
Genes and quantitative trait loci associated with schistosomiasis plotted on a human karyotype. Blue lines indicate QTL, with reported −log *p*-value for association shown on the y axis. Genes containing SNP associated with schistosomiasis infection (Table 3) are shown in blue, genes associated with pathology (Table 4) are shown in red and genes associated with both pathology and infection are shown in black. Genes are arranged vertically on the plot for clarity and their position on the y axis is arbitrary.

The *SM1* quantitative trait locus (QTL) on chromosome 5 5q31-q33 for *S. mansoni* fecal egg count was the first QTL to be mapped in humans for any infectious disease (Table 2, Figure 1) (4). The great success of this study was partially attributable to the very large effect size of the *SM1* locus (66% of the variance after accounting for water contact, age and sex). This is in striking contrast to the very modest proportions of the variance explained by most loci identified by GWAS in which loci rarely explain more than 10% of the variance of the trait that is not attributable to covariates (96). Three further loci (1p22.2, 7q36 and 21q22–22-qter) had evidence of association and contained genes known to be involved in the response to schistosomes but did not achieve genome wide significance (68). A reanalysis of the same data controlling for *SM1* genotype identified additional genome wide significant loci at 1p21-q23 and 6p21-q21 (Table 2, Figure 1) (69), since the effect of these loci was small in comparison to the effect of *SM1*, they were only identifiable when using *SM1* genotype as a parameter of the model.

**Table 2:** Loci associated with *S. mansoni* infection discovered by linkage studies.

**Phenotypes**	**Locus name**	**5' Marker (Position)**	**3'Marker (Position)**	**References**	**Candidate genes, Tested (Untested)**
Egg count	*SM1* 5q31-q33	D5S642 (128Mb)	D5S412 (158Mb)	(4, 68–70)	***IL4, IL5, IL9, IL13**, (IL3, CXCL14, CD14, 1IL17B, IL12B)*
Egg count	1p21-q23	D1S236 (95Mb)	D1S196 (168Mb)	(69)	*(IL6R, CRP)*
Egg count	6p21-q21	D6S271 (43Mb)	D6S283 (67Mb)	(69)	(*VEGFA, IL17A, IL17F*)
Hepatic fibrosis	*SM2* 6q22-q23	D6S1009 (137Mb)	D6S310 (142Mb)	(71)	***CTGF**, IFNGR1, IL22RA2*
Egg count	7q35-q36	D7S483 (152Mb)	D7S550 (156Mb)	(69)	(*TRB, NOS3, SHH)*

**Figure 2:**
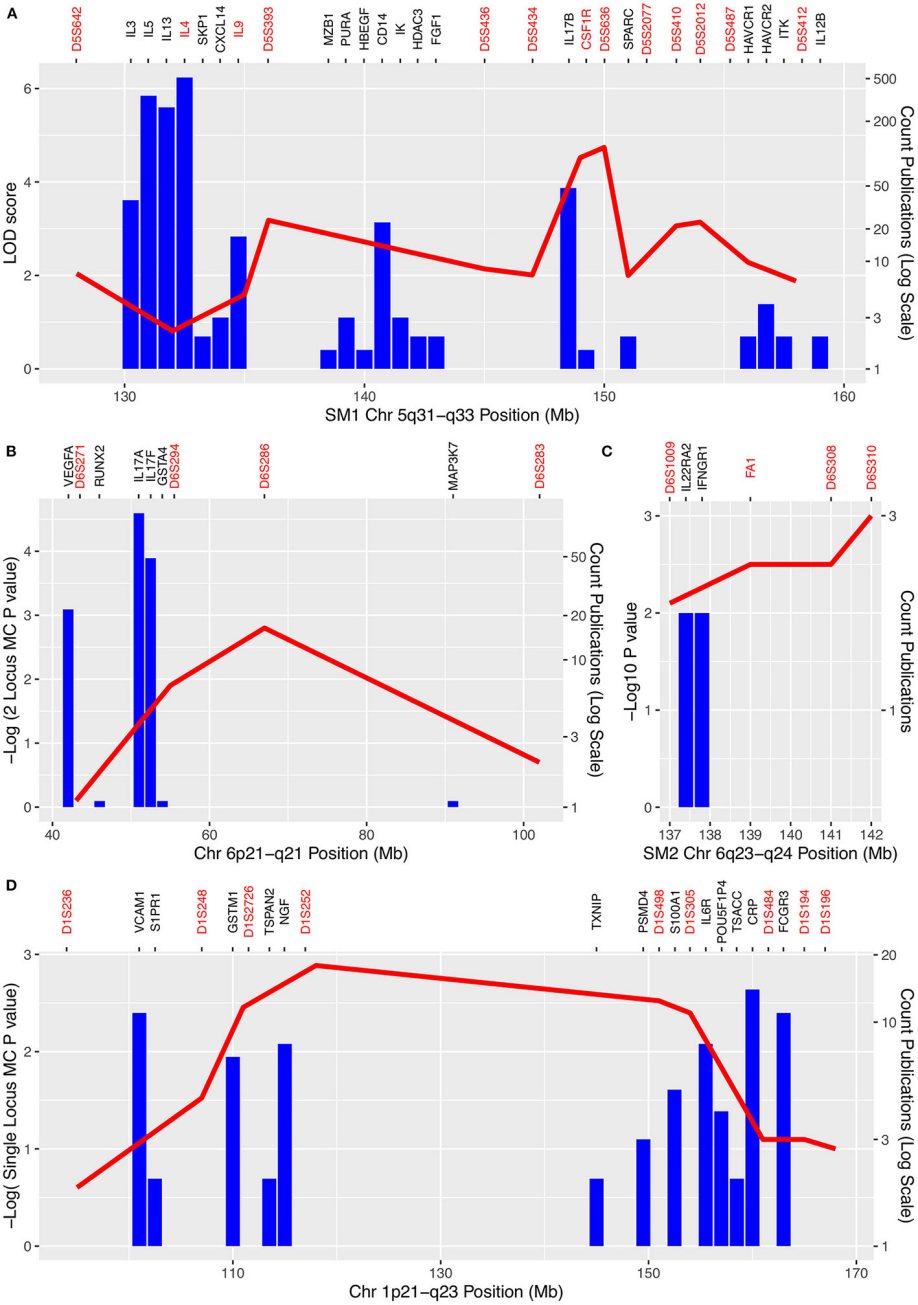
Schistosomiasis QTL and numbers of publications mentioning each gene in each QTL regions. **(A)** *SM1* region 5q31-q33; **(B)** 6p21-q21; **(C)** *SM2* 6q23-q24; **(D)** 1p21-q23. Negative log *p*-values for associations between markers and schistosomiasis are shown on the left-hand axis. Counts of publications which mention both schistosomiasis and genes in the QTL are shown by the blue columns and on a log scale on the right-hand y axis. Markers used in mapping are shown in red on the top axis (including genes used as markers). Genes for which there was only one publication are omitted for clarity and positions of genes and markers have been adjusted by up to 1Mb for clarity. Note the cluster of T_h_2 cytokine genes (*IL3, IL4, IL5, IL9, IL13*) in *SM1* with large numbers of publications between 131 and 135 Mb but low LOD scores. However, in a reanalysis of the same data using weighted pairwise correlations the peak of the QTL shifted toward this cytokine cluster (69).

*Marker positions are shown in GRCh37 co-ordinates. Candidate genes that have been found associated with the phenotype are shown in bold (Tables 3, 4). Other plausible candidates (See Figure 2) in the regions are in brackets. Genes associated with the T_h_17 response are underlined. These loci are shown graphically in Figures 1, 2*.

A further study on a Senegalese population, by the same group that conducted the original study in Brazil, confirmed an association at the *SM1* locus. However, the effect was not as strong and the association could only be demonstrated by non-parametric pedigree tests for an effect at the *SM1* locus (70). Non-parametric analysis requires no prior knowledge of the disease allele frequency or the disease penetrance of the different genotypes.

#### Linkage Studies Identify QTL for Pathology That Are Independent of QTL for Parasite Burden

A study in Sudan found that 12% of the study population had moderate or advanced fibrosis and that half of these also had portal hypertension (97). A linkage study of four candidate gene regions in the same population identified a locus (*SM2*) on chromosome six near the interferon-gamma receptor (*IFNGR1*) associated with hepatic fibrosis (Table 2, Figure 1) (98). Fifty percentage of people with risk alleles at *SM2* had some continuous thickening of periportal vein branches within 19 years of coming to live within the study area. IFNG is strongly anti-fibrogenic and polymorphisms in *IFNGR1* could plausibly regulate fibrosis. No linkage was found with the *SM1* locus suggesting that control of infection and pathology were independent. The *SM2* locus at 6q22-q23 did not overlap either the HLA region on chromosome six or the 6p21-q21 region that was associated with *S. mansoni* worm burden in Brazil (69) (Table 2, Figure 1).

The *SM2* locus was replicated in a linkage study of 11 candidate gene regions in Egypt where 32.7% of individuals 11 years and older had significant fibrosis and rs1327475 in *IFNGR1* was significantly associated with severe PPF. In contrast to the earlier study in Sudan, this study found a weak association with the T_h_2 cytokine cluster (IL4, IL13) in *SM1* (54), suggesting that worm burden does contribute to risk of pathology. There is evidence for a potentially protective role of a high IFNG response to schistosome infection, consistent with the anti-fibrogenic properties of IFNG [reviewed by Abath et al. (99)] and a SNP in *IFNG* has been associated with time to reinfection (Table 3) (72). Consequently, it appears that the variation in the IFNG system is involved in both the outcome of infection and pathology. Variation in *IFNGR1* has only been shown to be involved in the development of pathology but it has yet to be tested in a candidate gene study for effect on infection response.

**Table 3:** SNP which have been found to be associated with schistosomiasis infection phenotypes in candidate gene studies.

**SNP**	**Gene**	**Phenotype**	**Parasite**	**Neg refs**	**Pos refs**
rs2430561	*IFNG*	T2R	Sm	none	(72)
rs3024495	*IL10*	FEC	Sm	None	(38)*
rs1800896	*IL10*	IgE	Sm	None	(38)
rs1800871	*IL10*	IgE	Sm	None	(38)*
rs1800872	*IL10*	IgE	Sm	None	(38)*
IL10(-1082/-819/-592)	*IL10*	UEC	Sh	(73)	(74)*
rs20541	*IL13*	FEC	Sm	(75, 76)	(77)
rs2066960	*IL13*	FEC	Sm	(78)	(77)
rs7719175	*IL13*	UEC	Sh	(73)	(79)*
rs2069743	*IL13*	UEC	Sh	(78)	(80)
**rs1800925**	***IL13***	**UEC, FEC, T2R**	**Sh, Sm**	**None**	**(****72**, **77****–****79**)*
**rs2243250**	***IL4***	**UEC, T2R**	**Sh**	**(****80****)**	**(****73****)**, **(****72**)*
rs2079103	*IL5*	IF	Sj	None	(75)*
rs2706399	*IL5*	IF	Sj	None	(75)*
rs3024974	*STAT6*	UEC	Sh	None	(73)*
rs324013	*STAT6*	UEC	Sh	None	(78)*
rs733618	*CTLA4*	UEC	Sh	None	(81)
rs11571316	*CTLA4*	UEC	Sh	None	(81)
rs231775	*CTLA4*	UEC	Sh	None	(81)
rs3124952	*FCN2*	UEC	Sh	None	(82)
rs17514136	*FCN2*	UEC	Sh	None	(82)
rs7567833	*COLECC11*	UEC	Sh	None	(83)
COLEC11*TCCA	*COLECC11*	UEC	Sh	None	(83)
Blood group O	*ABO*	FEC,UEC	Sh,Sm	None	(84)
rs746822072	*RNASE3*	FEC	Sm	None	(85)

Loci that have been found associated with schistosomiasis in more than one study are shown in bold. * Loci that are not significant after Bonferroni correction. IL10 (−1082/−819/−592) = a haplotype of rs1800870, rs1800871 and rs1800872. COLEC11*TCCA is a haplotype of rs1864480 (−676T/C), rs4849953 (−472T/C), rs6714770 (−469C>G), and (−276C>T). Blood Group O is most commonly defined by genotypes at three SNP rs8176719, rs8176746(CC), rs8176747(GG). IF, Infection Frequency; T2R Time to Reinfection; UEC, Urine Egg Count; FEC, Fecal Egg Count: Sh, S. haematobium; Sm, S. mansoni; Sj, S. japonicum. “Pos refs” column contains citations for the studies that showed a significant association with phenotype and “Neg refs” column contains citations of studies that failed to show a significant association at that locus.

## Annotation of Genes in QTL Regions

Very few genes were known in the 5 QTL regions for schistosomiasis (Table 2) at the time that the QTL were discovered, and we are not aware of any attempts to identify the genes that are responsible for the QTL effect. Hundreds of genes are now known in these loci, each of which could potentially regulate the phenotype and we prepared a short list of the most likely candidates in each region. In order to discover which genes in each schistosomiasis QTL region might be involved in the response to schistosomiasis we used a custom Perl script to search PubMed with terms for schistosomiasis and each gene name and its aliases and obtained a count of the number of publications returned as detailed in Supplementary Table 4.

We assumed that the genes that are most likely to be the QTL genes will already have been shown to be involved in the response to schistosome infection. In order to identify these genes, we systematically searched PubMed for papers that included terms for schistosomiasis and each of the gene names in the 5 QTL regions in Table 2 or their synonyms (Supplementary Table 4). The genes that have been mentioned most frequently in the abstract of a paper that also mentions schistosomiasis and that are in one of the QTL are shown in Figure 2, Table 2. A complete list of all genes that are in the QTL and that have been studied in the context of schistosomiasis is in Supplementary Table 4. The number of papers shown in Figure 2 is an indicator of the genes most commonly associated with schistosomiasis in these regions. The genes with the largest literature were the T_h_2 cytokine genes originally identified by Marquet (4) in *SM1* (*IL3, IL4, IL5, IL*9, and *IL13*), that each had between 17 and 511 publications associated with schistosomiasis. Only *CSF1* and *TRB* (beta T cell receptor) were identified as candidate genes by the original authors in the 1p21-q23 and 7q35-q36 egg burden loci (69) and *IFNGR1* was the candidate gene that was used for the linkage study at *SM2* (98). The large literature on T_h_2 cytokines and schistosomiasis is expected given the important role of this pathway in response to egg antigens and the development of pathology. The T_h_2 cytokines in *SM1* are therefore credible candidate genes at this locus.

Our annotation of these QTL also revealed the presence of T_h_17 related genes in four of the five QTL: *IL17B* and *IL12B* in *SM1, IL22RA2* in *SM2, IL17A* and *IL17F* in 6p21-q21 and *IL6R* in1p21-q23. Although *IL12B* (*IL12p40*) in *SM1* is primarily known as a T_h_1 cytokine it is also a component of the hetero-dimeric IL23 cytokine which is important for T_h_17 maintenance and expansion (100) and IL6 is important in T_h_17 T helper cell differentiation (101). IL17 cytokines are involved in regulation of worm and egg burdens as well as the development of fibrosis and granuloma in response to eggs (102). The presence of T_h_17 related cytokines in four of the five QTL suggests that variation in this system may also contribute to variation in outcome of infection in addition to that caused by variation in the T_h_2 system.

## Candidate Gene Studies

### Infection Status and Intensity

We have identified 28 candidate gene studies of *Schistosoma* infection or worm burden that reported associations between 24 loci in eleven candidate genes and seven different phenotypes (Table 3, Supplementary Tables 2, 3). The genes with associations were *IFNG, IL10, IL13, IL4, IL5, STAT6, CTLA4, FCN2, COLECC11, ABO*, and *RNASE3*. These genes were all chosen because their protein products were known to be involved in the response to infection. One study of *MASP2* (103) and one on *LTA* (104) only reported negative results and are not included in Table 3. We have not attempted any formal meta-analysis since few loci were replicated and there were important differences in study design and data reporting in the studies of loci that were replicated, making any meta-analysis hard to interpret.

Schistosome eggs induce granulomatous T_h_2 responses (*IL4, IL5, IL9, IL10*, and *IL13*) (32) and antigen-specific IgG1, IgG4, and IgE (105). The *SM1* region on chromosome five identified by Marquet et al. (4) (Figure 1) included the prototypical T_h_2 cytokines *IL4, IL5, IL9*, and *IL13*, these are strong candidates for the QTL gene(s) and SNP and all except *IL9* have been found associated with schistosomiasis in candidate gene studies (Table 3). *IL13* and *IL4* regulate *STAT6* expression which in turn regulates IgE class switching (106) and *STAT6* variants are also associated with schistosomiasis (Table 3). *IL13, IL4, IL5*, and *STAT6* are also involved in regulation of the T_h_2 response to schistosomiasis (72, 75, 77–80).

T_h_1 cytokines and IFNG in particular are involved in the resistance to the immature worms. Studies of mice and *ex vivo* human PBMC have shown that IFNG levels increase in response to schistosome antigens and are correlated with resistance or tolerance to infection (27, 107, 108) and a candidate gene study found an association between the *IFNG* SNP rs2430561 and time to reinfection (72). IL10 and CTLA4 downregulate immune responses in long standing infections (31). *COLECC11* and *FCN2* are involved in the innate immune response, they both bind to specific pathogen-associated molecular patterns (PAMPs) on the pathogen surface and stimulate the complement lectin cascade, thereby clearing the pathogens by opsonization (82, 83). *ABO* regulates blood group and a meta-analysis found evidence for a protective effect for blood group O (84). RNASE3 also known as eosinophil cationic protein (ECP) is a secretory protein of eosinophil granulocytes that efficiently kills the larval stage of *S. mansoni* (85).

The Ensembl Variant Effect Predictor was used to provide functional annotations for these variants (Supplementary Table 5). In the functional annotation only rs231775 in *CTLA4* and rs20541 in *IL13* were predicted to have an effect on function. Both of which were non-synonymous variants and were classified as risk factors by ClinVar (109), although SIFT (110), and Polyphen (111) predicted the effect of these SNP would be benign. Other SNP had no predicted effects on function, possibly because they are not functional but are linked to functional SNP nearby. However, the functional annotation cannot detect all functional variants and experimental work has shown that *IL13* expression is regulated by rs1800925 (112). Further detailed studies will be required to determine which of the SNP are truly functional and which are not functional but still potentially useful markers for risk of schistosomiasis.

### Pathology: Hepatic and Periportal Fibrosis

It has been noted since at least 1974 that the development of severe fibrosis is clustered in families and is not well-correlated with intensity of infection suggesting that the mechanisms regulating infection intensity and pathology are not closely coupled (5). We found 20 studies which identified associations with schistosomiasis related pathology at 46 candidate SNP or haplotypes in 21 genes outside the HLA complex (Table 4, Supplementary Table 2). Few of the studies applied any multiple testing corrections and 15 out of the 43 associations would not be significant after a Bonferroni correction (Table 4).

**Table 4:** SNP which have been found to be associated with schistosomiasis pathology related phenotypes in candidate gene studies.

**SNP**	**Gene**	**Phenotype**	**Species**	**Not associated**	**Associated**	**References**
rs1327475	*IFNGR1*	HF	Sm	NA	Egypt	(54)
rs2243250^§^	*IL4*	HF	Sm	NA	Egypt	(54)
rs1056854	*TGFB1*	HF	Sm	NA	Egypt*	(54)
rs373880612	*IFNG*	PPF, HF, PH	Sm	NA	Sudan*	(55)
rs1861494	*IFNG*	PPF, HF, PH	Sm	NA	Sudan*	(55)
IFN-gR1 6q22-q23	*IFNGR1*	HF, PH	Sm	NA	Sudan	(71)
rs3037970	*CCN2(CTGF)*	HF, PH	Sj Sm	China Sudan Brazil	China	(71)
rs1257705	*CCN2(CTGF)*	HF, PH	Sj Sm	Sudan Brazil China	China*	(86)
rs2151532	*CCN2(CTGF)*	HF, PH	Sj Sm	China Egypt	China*	(54, 86)
**rs9402373**	*CCN2(CTGF)*	HF, PH	Sj Sm	NA	2 China* Sudan Brazil*	(86)
rs9399005	*CCN2(CTGF)*	HF, PH	Sj	China	China*	(86)
rs6918698	*CCN2(CTGF)*	HF, PH	Sj Sm	China Sudan	China* Brazil*	(86)
rs1931002	*CCN2(CTGF)*	HF, PH	Sj	China	China	(86)
**rs12526196**	*CCN2(CTGF)*	HF, PH	Sj Sm	Sudan Brazil	2 China*	(86)
rs1800925C-rs20541A^§^	*IL13*	HF, PH	Sj	NA	China	(87)
rs1800925^§^	*IL13*	HF PH	Sj	NA	China*	(87)
**rs12712135**	*ST2 (IL1RL1)*	IL1RL1	Sj Sm	NA	China Brazil	(88)
**rs1420101**	*ST2 (IL1RL1)*	IL1RL1	Sj Sm	NA	China Brazil	(88)
**rs6543119**	*ST2 (IL1RL1)*	IL1RL1	Sj Sm	NA	China Brazil	(88)
rs7412, rs429358 APOE3	*APOE*	TC, LDL	Sm	Brazil	NA	(89)
**rs6570136**	*IL22RA2*	HF, PH	Sj Sm	NA	China Brazil* Sudan*	(90)
**rs7774663**	*IL22RA2*	HF, PH	Sj Sm	China	Sudan Brazil*	(90)
**rs2064501**	*IL22RA2*	HF, PH, IL22	Sj Sm	NA	China* Brazil* Sudan*	(90)
rs11154915	*IL22RA2*	HF, PH, IL22	Sj Sm	China Brazil	Sudan*	(90)
rs7749054	*IL22RA2*	HF, PH, IL22 level	Sj Sm	China Sudan	Brazil*	(90)
rs1800870	*IL10*	PPF	Sm	NA	Brazil	(91)
IL10 (-1082/-819/-592)^§^	*IL10*	PPF	Sm	NA	Brazil	(91)
rs1800629	*TNFA*	PPF, TNFA	Sm	NA	Brazil	(92)
**rs10118570**	*MAPKAP1*	HF, infection	Sj	NA	2 China	(93)
rs391957	*HSPA5*	HF, infection	Sj	NA	China	(93)
rs17025963 rs185882198 rs71093915	*TGFBR2*	HF	Sm	NA	Brazil	(5)
rs1690215 rs56368010	*ACVRL1(ALK1)*	HF	Sm	NA	Brazil	(5)
rs114046193 rs12345675	*TGFbR1 (ALK5)*	HF	Sm	NA	Brazil	(5)
rs138079455 rs10555873 rs77414361	*SMAD9*	HF	Sm	NA	Brazil	(5)
rs12913547 rs12914140 rs12439500	*SMAD3*	HF	Sm	NA	Brazil	(5)
rs746822072	*RNASE3 (ECP)*	HF	Sm	NA	Uganda	(85)
rs1800629	*TNFA*	PPF regression after treatment	Sm	NA	Brazil	(94)
rs2295080	*mTOR*	HF, infection	Sj	NA	China	(95)
rs7254617	*AKT2*	HF, infection	Sj	NA	China*	(95)

Loci that have been found associated with schistosomiasis in more than one population are shown in bold. *Loci that are not significant after Bonferroni correction. ^§^SNP which are also associated with infection (Table 3). Studies of classical HLA loci have been excluded since they have been fully reviewed elsewhere (7, 8). Sh, S. haematobium; Sm, S. mansoni; Sj, S. japonicum. The “Associated” column shows the locations of studies where a significant association has been found and the “Not Associated” column shows the location of studies that failed to show a significant association. PPF Periportal Fibrosis; PH Portal Hypertension; PT Portal thickness, including portal vein diameter and portal vein branch point thickness, HF Hepatic Fibrosis; AHSD Advanced HepatoSplenic Disease, TC Total Cholesterol, HDL High Density Lipoprotein, TNFA Median levels TNFA higher in patients with PPF, IL22 level of IL22 in plasma, IL1RL1 level of soluble IL1RL1 in plasma. IL-10 (−1082/−819/−592) represents a haplotype of rs1800870, rs1800871, and rs1800872. NA, Not applicable.

There are sixteen genes for which an effect has only been reported for fibrosis and not for infection: *APOE, CCN2, HSPA5, IFNGR1, IL22RA2, MAPKAP1, IL1RL1, TNFA*, mTOR, AKT2, *TGFB1, TGFBR1, TGFBR2, ACVRL1, SMAD9*, and *SMAD3* (Tables 3, 4). Five genes have been associated with both fibrosis and intensity of infection (*RNASE3, IL4, IL10, IFNGI*, and *IL13*). Four genes were associated with fibrosis in more than one population: *IFNGR1, IL22RA2, CCN2*, and *MAPKAP1* (Table 4), the two former genes are also in the *SM2* QTL (55, 71, 86, 90, 93). Although these sixteen genes have not been tested for associations with infection status or intensity it is plausible that some of them are only associated with pathology. All of these genes except *APOE* and *IL1RL1* have been associated with the regulation of fibrosis (Table 4) and variation in these may regulate risk of pathology irrespective of intensity of infection.

The Ensembl Variant Effect Predictor was used to provide functional annotations for the SNP in Table 4 (Supplementary Table 5). Two non-synonymous SNP in *APOE* (rs7412 and rs429358) were predicted by ClinVar to be a risk factor, pathogenic and involved in drug response (109), the rs7412 SNP was also predicted to be deleterious or damaging by SIFT (110) and Polyphen (111). A non-coding SNP in *LTA* (rs1800629) was predicted to be involved in drug response by ClinVar (109) and a non-coding SNP (rs1800872) in *IL10* was predicted to be risk factor by ClinVar. Other SNP in Table 4 did not have functional annotations, and many may be marker SNP that are linked to functional variants rather than functional themselves.

A recent study found that just 32 SNPs could predict who gets severe hepatic fibrosis in Brazil with 63% sensitivity and 90% specificity (5). This review emphasized the importance of TGFB signaling pathway and IL22. TGFB is also involved in the differentiation of T_h_17 cells (113), and together with SMAD regulates T_h_17 in response to another worm infection *Echinococus multilocularis* (114) providing further justification for systematic investigation of the role of variants in the T_h_17 pathway in differences in response to infection. IL22 and IL17 are co-expressed by T_h_17 CD4+ T cells and polymorphisms have been associated with hepatic fibrosis in the *IL22* receptor *IL22RA2* (5, 90). IL22 also has protective effects on the intestinal epithelium against toxic bacterial products (5).

### Associations With the HLA Region

The HLA region is associated with response to many communicable and non-communicable diseases. The importance of CD4+ T helper cells in the response to schistosomiasis, and the role of HLA class II alleles in recruiting these, suggests that variation in the HLA region may play an important role in control of schistosome infections. However, associations were not found in this region in the whole genome linkage scans either for worm burden or pathology (4, 68, 71).

Two reviews reported 17 and 18 studies, respectively, of associations of HLA markers with schistosomiasis induced PPF (7, 8), but surprisingly we could not find any studies of HLA genes and worm burden (Table 3, Supplementary Table 3). We have found only one study of genes in the HLA region and schistosomiasis that has been published in the 9 years since those reviews. A SNP in Major histocompatibility complex class I chain-related A (*MICA*) was associated with liver fibrosis in a Han Chinese population (76).

It has been emphasized that the problems of extensive linkage disequilibrium within the HLA region, the small sizes of the studies reviewed, the allelic diversity and large variations in allele frequencies between populations mean that these studies may not replicate in different populations and need further confirmation from larger studies (7). However, some alleles of HLA class II loci DQA1, DQB1 and DRB1 and HLA class I HLA-A and HLA-B were associated with PPF in a meta-analysis that combined evidence from 2 to 3 studies for each allele evaluated (8) and these associations may be robust.

## Discussion

### Replication of Candidate Gene Studies for Infection

Few of the candidate gene studies shown in Tables 3, 4 applied correction for testing multiple SNPs and the 32 associations that would not remain significant after such a correction are indicated by asterisks. The lack of Bonferroni corrections suggests that some of these studies will not replicate and, for some of these loci, there are studies that have not replicated the association (Tables 3, 4), although this is often when using a different phenotype. Notably, half of these studies that did not replicate an association were at loci that were significant after a Bonferroni correction.

There are many instances of failures to replicate candidate gene studies. One review found that only 6 out of 166 associations replicated in more than 75% of studies, although 97 of the 166 associations (58%) were reproduced in at least one study (115). Failure to replicate can be due to the initial observation being due to random variation in allele frequencies between test and control samples (a type one error). However, genuine associations can fail to replicate because of linkage between the marker SNP and the functional SNP varying between populations, small study sizes, variable penetrance, population variation at modifier loci, (occult) population stratification within study populations or differences in allele frequencies between populations leading to type two errors. In addition, it is also possible for different SNP in the same gene to be most important in regulating a response in different populations or individuals (116). Therefore, most of these observations should be considered provisional until adequately powered meta-analyses can be conducted.

Associations with infection were replicated at two SNP (*IL13* rs1800925; *IL4* rs2243250), and both of these SNP and also the *IL10* (−1082/−819/−592) haplotype were also found to be associated with pathology (Tables 3, 4), despite the studies using different phenotypes and in one case different parasite species. The *IL13* rs1800925 SNP was associated with schistosomiasis in four infection related studies and one pathology study and all studies that included this SNP found an association with it, despite the association not being significant after a Bonferroni correction in any of these studies. The high level of replication at this SNP suggests that these associations may be robust despite the lack of significance in individual studies. Functional data also supports a role for rs1800925, which is in the promoter of *IL13*, and is associated with increased expression of IL13 from stimulated cells *in vitro* (112). Since IL13 regulates IgE levels via STAT6 (106), there is a plausible mechanism for a role for this SNP in response to infection, increasing the confidence that it is a genuine association. Although *IL4* expression is also associated with IgE levels, the rs2243250 SNP is not (117), so its impact on intensity of infection must be via some other mechanism.

### The Th17 Pathway Has Been Neglected in Schistosomiasis Association Studies

Since the Th2 pathway is the dominant response to helminth infections and is the main pathway for response to egg antigens (28, 30), genes in this pathway have been well-represented in association studies (Tables 3, 4) and these have confirmed the importance of variation in this pathway for outcome of infection. However, the Th1 and Th17 pathways are also important, particularly in the early stages of the infection (26, 29, 36, 37). Variants in *IL17F* and *IL17RA* have been found associated with cerebral malaria (118) and similar variation may contribute to the outcome of schistosome infection. Our annotation of QTL, with the genes that have published associations with schistosomiasis, revealed an excess of Th17 pathway genes in these QTL (Figures 1, 2). There have been no association studies to test candidate gene hypothesis for three out of the five QTL (Table 2) and there are Th17 genes in four of the five QTL (underlined in Table 2), which could be priorities for future association studies.

It is possible that variation in other Th17 pathway genes outside of the QTL also contribute to variation in response to infection. A KEGG pathway diagram of Th17 cell differentiation and a list of 108 genes in this pathway is shown in Supplementary Table 6. We have obtained a list of the 1,742,019 SNP in these genes from dbSNP and kept the 1,052 SNP that were predicted to be “pathogenic” by ClinVar, irrespective of minor allele frequency (Supplementary Table 7). We also kept SNP with minor allele frequency > 5% and that had any of the following functional classifications: splice acceptor variant, stop gained, initiator codon variant, stop lost, splice donor variant, missense variant, terminator codon variant, frameshift variant. This left a list of 2,701 SNP in the Th17 pathway which are most likely to have an effect on function and that could be priorities for further testing (Supplementary Table 7).

### Which Are the Optimal Study Designs to Discover Susceptibility Loci?

Approaches for discovering susceptibility loci for parasitic infections have been reviewed previously (119). The major approaches are association studies in unrelated individuals and linkage studies within families, the merits of which have been evaluated by Abel and Dessein (120). We noted in this review that family based designs were the first to discover QTL loci in schistosomiasis (4), and these were followed up with considerable success by candidate gene studies in these QTL. Schistosomiasis affected communities are frequently geographical clusters of related individuals where case control studies can be confounded by cryptic relatedness. In contrast family-based association studies exploit this relatedness by estimating disequilibrium in transmission of alleles within families.

Schistosomiasis is also an excellent setting for family-based linkage studies of infection intensity because children are the most heavily infected; therefore, parents are often available for genotyping to create complete families, unlike adult onset diseases. However, it is more difficult to collect full families for complications of chronic schistosomiasis such as fibrosis that affect adults. Whole genome linkage studies have only been undertaken on two populations, one in Brazil and one in Senegal, further studies to identify loci regulating intensity of infection in additional populations should be undertaken and could enlarge our understanding of the mechanisms of response to infection. It has already been shown that 32 SNP can be used to identify those at highest risk of developing pathology after *S. mansoni* infection (5). If the 20% of the people that shed 80% of the eggs could also be identified they could be targeted for regular treatment which could dramatically reduce the number of eggs in the environment and the pressure of infection on the whole community.

## Conclusion

Despite the remarkable success of the early linkage studies that identified major QTL loci, no further whole genome scans for association have been conducted and the QTL genes underlying these loci have not been definitively identified. All subsequent studies have been candidate gene linkage and association studies focussing on genes within the QTL regions *SM1, SM2*, and the T_h_2 pathway that are hypothesized to play a role in schistosomiasis progression. No candidate gene studies have attempted to identify QTL genes in three of the QTL for *S. mansoni* egg count (Table 2). This review has presented evidence that the T_h_17 pathway has been overlooked in studies of the genetics of schistosomiasis and should be prioritized in future investigations of susceptibility genes.

The rapid development of genotyping technologies makes large scale genomic studies easier than ever, provided that well-characterized samples can be obtained. The studies of Dessein et al. (86) on populations from China, Sudan and Brazil have demonstrated the value of replicating analyses in multiple populations and similar replication is needed for other candidate SNP and genes.

## Author Contributions

GS and EMa conceived the review. EMe and ON wrote the draft and collected all the relevant literature. HN significantly reviewed the draft and analyzed QTL data. ME contributed to the draft and revised the manuscript. All authors read and approved the final manuscript.

## The TrypanoGEN+ Research Group of the H3Africa Consortium

Membership of the TrypanoGEN+ research group is available at http://www.trypanogen.net/.

## Conflict of Interest

The authors declare that the research was conducted in the absence of any commercial or financial relationships that could be construed as a potential conflict of interest. The handling editor declared a past co-authorship with the authors HN, EMa, and GS.

## Supplementary Material

Supplementary Table 1 Search strategy: Summary of PubMed search strategy.

Supplementary Table 2 All included references: Papers included in the review.

Supplementary Table 3 Details of Refs Populations, study design, phenotypes, and effects from studies.

Supplementary Table 4 Genes in QTL. List of genes in QTL regions with lists of publications associated with each gene and schistosomiasis.

Supplementary Table 5 VEP SNP Annotations. Functional annotation of SNP from candidate genes studies with the Ensembl Variant Effect predictor.

Supplementary Table 6 Th17 genes. List of Genes in Th17 pathway with KEGG pathway diagram.

Supplementary Table 7 Th17 SNP. List of potentially function SNP in genes in the Th17 pathway.
